# An Efficient Detection Platform Based on Mesoporous Au@Cr_2_O_3_ Particles with Schwarz P Surface for Precise Periodontitis Metabolite Profiling

**DOI:** 10.1002/advs.76441

**Published:** 2026-07-27

**Authors:** Yue Sun, Yan Wang, Fangying Shi, Wenhe Xie, Yiding Zhang, Heyuhan Zhang, Chunhui Deng, Limin Wu, Meihua Chen, Yonghui Deng

**Affiliations:** ^1^ Department of Chemistry Shanghai Stomatological Hospital & School of Stomatology State Key Laboratory of Coatings for Advanced Equipment Shanghai Key Laboratory of Molecular Catalysis and Innovative Materials Fudan University Shanghai P. R. China; ^2^ School of Materials Science and Chemical Engineering Ningbo University Ningbo P. R. China; ^3^ Institute of Energy and Materials Chemistry Inner Mongolia University Hohhot P. R. China

**Keywords:** matrix‐assisted laser desorption/ionization mass spectrometry, mesoporous materials, metabolic fingerprinting, metal oxide matrix, periodontitis

## Abstract

Matrix materials with high desorption and ionization efficiency are crucial for the sensitive and accurate metabolite profiling in matrix‐assisted laser desorption ionization mass spectrometry (MALDI‐MS). Herein, we develop an efficient detection platform based on Au nanoparticles decorated mesoporous Cr_2_O_3_ (Au@mCr_2_O_3_) particles with a single primitive continuous structure (i.e., Schwarz P surface) as the matrix. Owing to the low‐tortuosity mesoporous pathways and customized chemical composition, the Au@mCr_2_O_3_ exhibits high‐efficiency light absorption and electron transfer, showing great potential for sensitive, reproducible, and selective metabolite detection. Therefore, it is considered as a high‐performance and reliable matrix material for metabolic biomarkers screening. As a proof of concept, the Au@mCr_2_O_3_‐based detection platform decodes the saliva metabolic fingerprints. With the assistance of machine learning, the platform achieves high diagnostic performance for periodontitis, with the areas under the curve of 0.875–0.934. Furthermore, a simplified biomarker panel is established, which provides a deeper mechanism understanding of the metabolic alternation of periodontitis and offers new insights into its diagnosis and pathogenesis.

## Graphical abstract

A non‐invasive periodontitis diagnosis platform was developed using Au nanoparticles‐decorated mesoporous Cr_2_O_3_ (Au@mCr_2_O_3_) particles with Schwarz P surface as matrix for saliva metabolic fingerprinting (SMFs) analysis via MALDI‐MS. With the assistance of machine learning of SMFs, this platform enables efficient diagnosis and the screening of potential biomarkers, thus establishing a simplified diagnostic model and elucidating the possible mechanism about periodontitis pathogenesis.

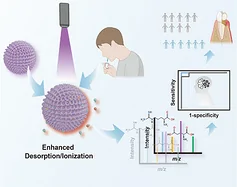

## 1 Introduction

As one of the most common chronic inflammatory diseases, periodontitis affects over 40% of adults, and its prevalence is on the rise worldwide, causing a considerable economic and public health burden [1, 2, 3]. Periodontitis is mainly caused by bacterial infections and dysbiosis of the periodontal microbial community [4]. It is the primary cause of tooth loss in adults and is closely related to numerous systemic diseases such as cardiovascular diseases, Alzheimer's disease, diabetes, and cancer [5, 6, 7]. The clinical diagnosis methods for periodontitis (e.g., palpation, probing depth measurement, radiography) are usually labor‐intensive, costly, and time‐consuming, making it difficult to monitor the disease progression dynamically and provide effective guidance for targeted treatment [8, 9]. Therefore, there is an urgent need to develop rapid and accurate diagnostic strategies for periodontal disease to alleviate the health burden.

Metabolic alterations are faithful real‐time reporters of cellular pathophysiology and presymptomatic disease [10, 11, 12, 13]. Therefore, decoding metabolic information is a reliable approach to promoting early disease diagnosis and guiding precise therapy [14, 15, 16]. As an ideal diagnostic medium, saliva offers abundant pathophysiological information for disease decoding, facilitating the realization of non‐invasive, safe, and painless sampling [17, 18]. Among various analysis platforms, matrix‐assisted laser desorption ionization mass spectrometry (MALDI‐MS) has gained widespread application in biofluid metabolic analysis due to its high throughput, operation simplicity, and rapid detection capabilities [19, 20, 21]. Currently, the widely used organic matrices in MALDI‐MS suffer from problems such as high background intensity, poor stability, and inhomogeneous co‐crystallization with analytes, which are prone to generating interference fragments and obstructing low‐molecular signals [22, 23]. As a result, the metabolic profiling of periodontitis through saliva has not yet been fully explored due to the lack of powerful matrix that is capable of overcoming the complex salivary environment, the low abundance of small‐molecule metabolites, and the ion suppression caused by high‐salt and high‐protein solutions. The development of advanced inorganic matrix is expected to achieve sensitive detection of metabolites and accurate monitoring of health status by MALDI‐MS.

Recently, metal oxide semiconductors (MOSs) are considered as promising candidates for inorganic matrix materials due to their outstanding thermal stability, photothermal and photoelectric properties [24, 25]. Nevertheless, the high recombination rate of photogenerated carriers still limits their ionization efficiency [26, 27, 28]. Introducing co‐catalysts such as Au nanoparticles (NPs) to customize the chemical microenvironment of MOSs is an effective strategy to suppress the carrier recombination and enhance the photothermal conversion [29, 30, 31]. Besides, to satisfy the high demand for high‐accuracy detection of periodontitis metabolites, it is necessary to rationally design the nanoarchitectures of MOSs to maximize their light absorption and mass transfer efficiency [32]. Single primitive continuous structure with a Schwarz P surface, also known as plumber's nightmare network, is an ideal topological structure for photonic and catalytic applications [33, 34, 35]. The low‐tortuosity mesoporous pathways within the structure can promote the harvesting of light reflection through multireflection and the fast diffusion of analytes, and the continuous semiconducting framework can facilitate the transportation of carriers [36, 37]. However, the application of inorganic materials with a Schwarz P surface in metabolic detection has rarely been reported due to the lack of effective synthesis methods.

In this work, Au NPs‐decorated mesoporous Cr_2_O_3_ (Au@mCr_2_O_3_) particles with a Schwarz P surface were synthesized and employed as a matrix to construct a high‐performance metabolite detection platform. First, polymer cubosomes (PCs) with inverse bicontinuous structure (*Im* $\bar{3}$ *m* symmetry) were prepared as template through the self‐assembly of amphiphilic block copolymers. Then, the open channels of PCs were selectively utilized for the confined crystallization of metal oxides, and mCr_2_O_3_ with a Schwarz P surface (*Pm* $\bar{3}$ *m* symmetry) can be obtained after the removal of templates via simple calcination. Finally, Au NPs were deposited on the pore wall of the mCr_2_O_3_ through an impregnation‐reduction process. Owing to its abundant active sites, high laser adsorption efficiency, low carriers combination rate, and excellent co‐crystallization ability, the Au@mCr_2_O_3_‐based MALDI‐MS detection platform exhibited outstanding sensitivity, reproducibility, and selectivity for metabolite analysis in a high‐salt and high‐protein environment. The established platform can rapidly extract the saliva metabolic fingerprints (SMFs) of each sample within only 10 s and achieve accurate diagnosis (area under the curve (AUC) of 0.875–0.934) under the assistance of machine learning algorithms (Scheme 1). Six potential metabolic features were successfully identified, providing a preliminary biomarker panel for periodontitis discrimination. Based on this panel, the detection platform further demonstrated favorable performance in blind tests with an AUC of 0.981–1.000.

**SCHEME 1:**
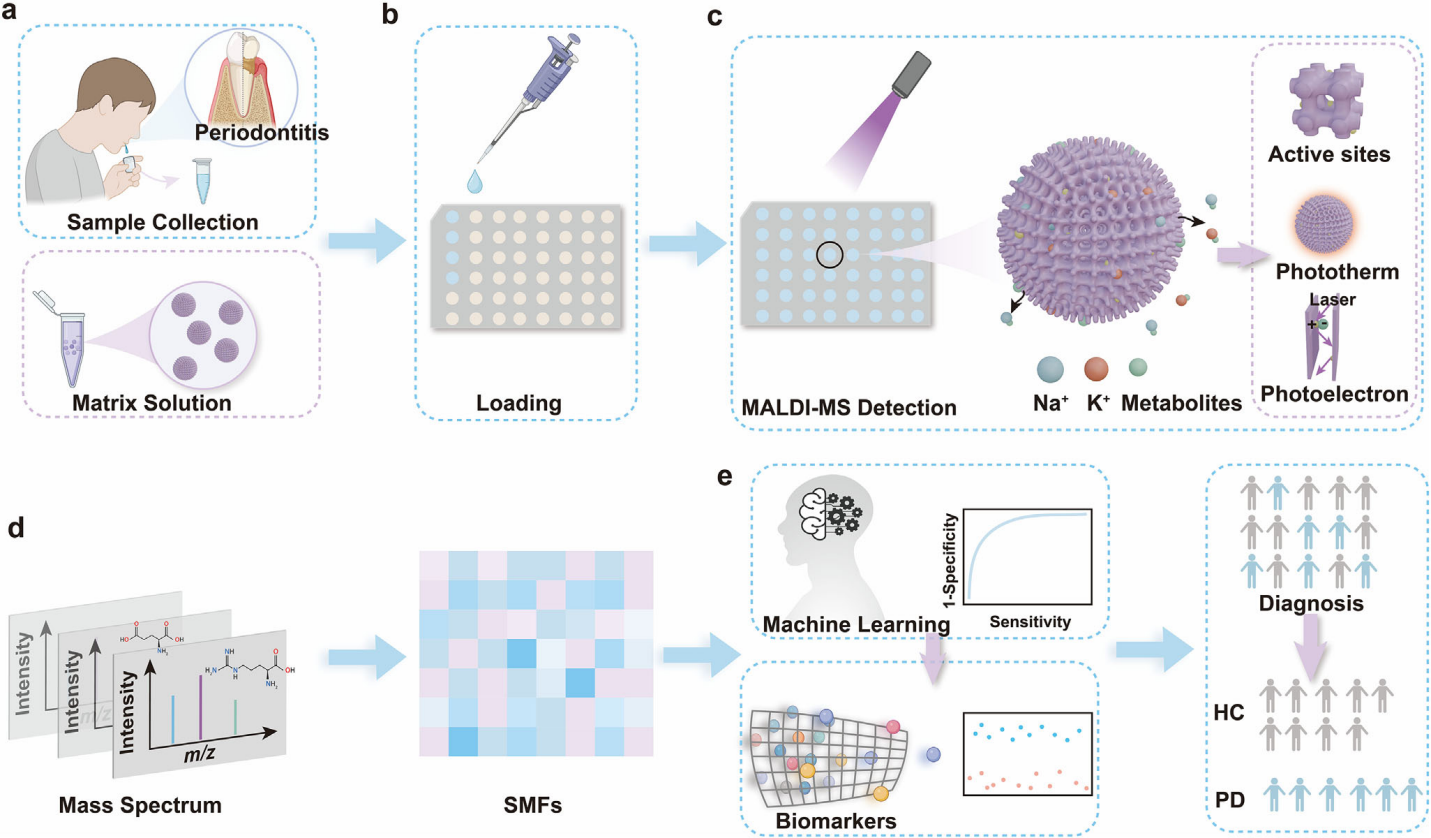
Schematic illustration of the detection and analysis of SMFs using Au@Cr_2_O_3_‐based MALDI‐MS. Workflow of the detection process via Au@Cr_2_O_3_‐based MALDI‐MS, including (a) periodontitis saliva sample collection and preparation of the matrix solution, (b) sample loading, (c) metabolites detection under the assistance of matrix materials. Workflow of the data processing, including (d) feature extraction, (e) machine learning‐assisted diagnosis, biomarker identification, and biomarker‐assisted classification for periodontitis.

## 2 Results and Discussion

### 2.1 Synthesis and Characterization of Au@mCr_2_O_3_

The synthesis procedure of Au@mCr_2_O_3_ is illustrated in Figure 1a. First, PCs are prepared through the self‐assembly of amphiphilic diblock copolymer poly(ethylene oxide)‐*block*‐polystyrene (PEO‐*b*‐PS). Under the induction of hydrophobic interaction, the PEO‐*b*‐PS progressively assembles from micelles into complex vesicles, and ultimately into PCs to minimize the interface tension. Scanning electron microscopy (SEM) and transmission electron microscopy (TEM) images reveal that the PCs have spherical morphology with perforated surface and bicontinuous mesoporous channels (Figure 1b; Figure S1a, b), in which only one set of the continuous mesoporous channel is connected to the external environment. Small‐angle x‐ray scattering (SAXS) pattern shows a set of peaks corresponding to inverse bicontinuous structure (*Im* $\bar{3}$ *m* symmetry), which is in consistence with the SEM characterization (Figure S1c). After that, under the assistance of capillary force, the open channels of PCs are selectively filled by Cr(NO)_3_ precursor, followed by the confined crystallization of Cr_2_O_3_ through thermal treatment. After removing the PCs templates via calcination, mCr_2_O_3_ particles with continuous mesoporous structure and Schwarz P surface were obtained. SEM and TEM images reveal that the mCr_2_O_3_ particles inherit the spherical morphology and ordered mesoporous structure of PCs (Figure 1c; Figure S2). SAXS pattern confirms the single primitive topology of mCr_2_O_3_ with a unit cell parameter of 79.5 nm, which is consistent with the SEM result, indicating the successful replication of the pore structure of PCs (Figure 1f). Nitrogen adsorption–desorption isotherms of the mCr_2_O_3_ show the type‐IV isotherm with a H_1_‐type hysteresis loop, indicative of a high specific surface area of 75.2 m^2^/g and large mesopores of ∼39 nm, which is beneficial for the diffusion and adsorption of analytes (Figure S3). The ordered continuous mesoporous structure of mCr_2_O_3_ with large mesopores and low‐tortuosity network enables analytes and laser to more easily enter the deep interior of nanochannels, thereby increasing the accessible active sites and enhancing the energy transfer efficiency. High‐resolution transmission electron microscope (HRTEM) image and selected‐area electron diffraction (SAED) pattern indicate the polycrystalline feature of mCr_2_O_3_ particles (Figure 1d). Energy dispersive x‐ray (EDX) element mapping reveals the homogeneous distribution of Cr and O elements throughout the mCr_2_O_3_ particles (Figure 1e). X‐ray photoelectron spectroscopy (XPS) analysis is conducted to investigate the elemental composition and valence states of mCr_2_O_3_. Cr 2*p* spectrum shows the coexistence of mixed valence states of Cr^3+^ and Cr^6+^. Similarly, O 1*s* spectrum displays that there are abundant oxygen vacancies in mCr_2_O_3_, which may facilitate the charge transfer in MALDI‐MS and enhance the energy utilization of the laser (Figure S4) [38].

**FIGURE 1:**
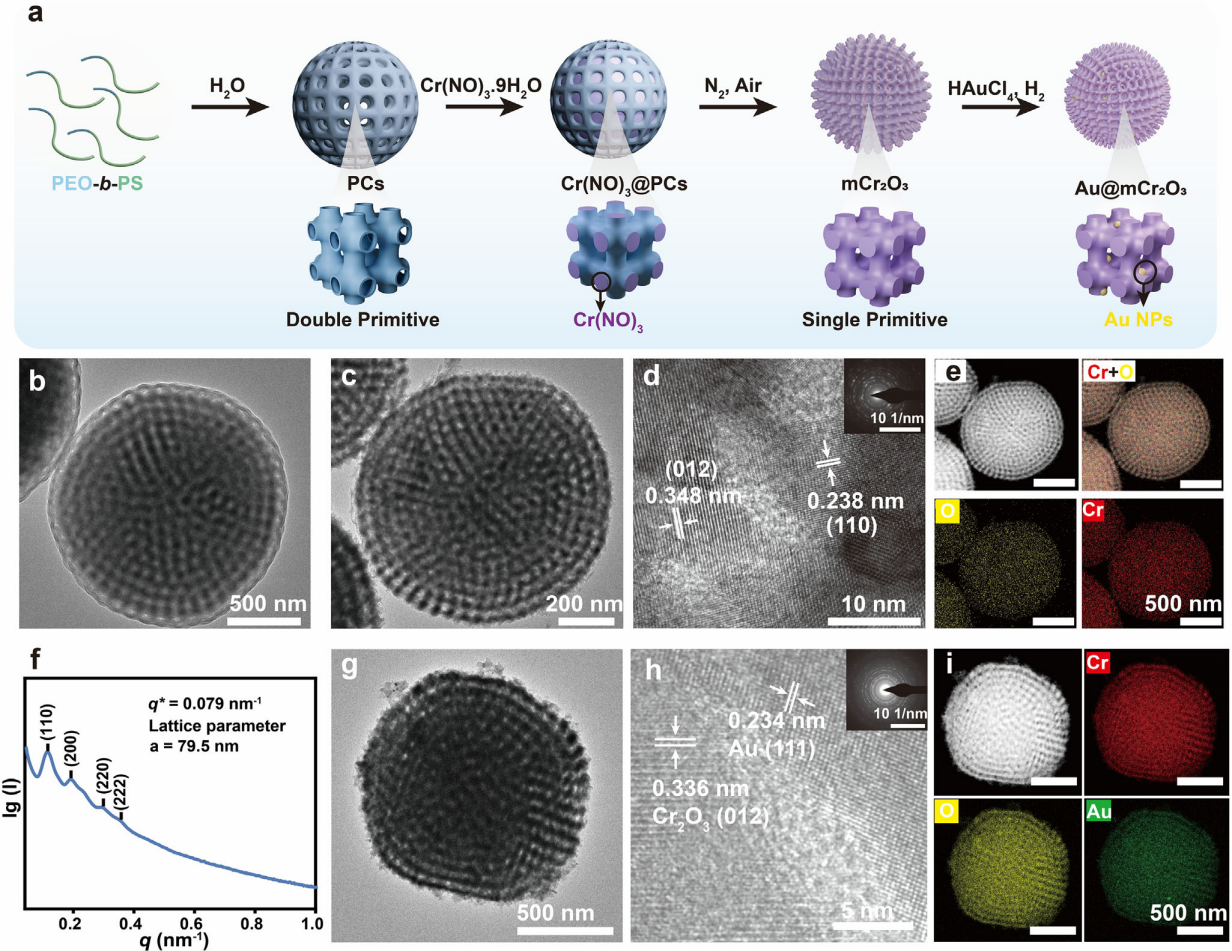
Synthesis and characterization of mCr_2_O_3_ and Au@mCr_2_O_3_ particles. (a) Schematic illustration of the synthesis of Au@mCr_2_O_3_ using PCs as templates. (b, c) TEM images of PCs (b) and mCr_2_O_3_ (c). (d–f) HRTEM image (d), EDX element mapping (e), and SAXS pattern (f)M, lattice parameter *a*  = 2π/q* ) of mCr_2_O_3_. (g–i) TEM image (g), HRTEM image (h), and EDX element mapping (i) of Au@mCr_2_O_3_‐1.0. The insets in (d) and (h) are corresponding SAED patterns.

As the photothermal hotspots and electron mediators, Au NPs are introduced as co‐catalysts to suppress the carrier recombination and enhance the photothermal conversion [39, 40, 41]. After a simple impregnation‐reduction process, Au@mCr_2_O_3_‐n (n = 0.5, 1.0, 3.0, referring to the theoretical weight percentage of Au NPs) particles are obtained, and the amount of Au NPs can be adjusted by changing the feeding amount of HAuCl_4_ precursor. TEM image and SAXS pattern show that the Schwarz P surface of mCr_2_O_3_ is well‐retained after the decoration of Au NPs, while the mesoporous skeleton slight deforms due to structure shrinkage during calcination treatment (Figure 1g; Figure S5a). HRTEM image clearly shows the (111) lattice plane of Au, confirming that the Au NPs have been successfully deposited in the mCr_2_O_3_ framework (Figure 1h). EDX element mapping exhibits the homogeneous distribution of Au throughout the mesoporous skeleton (Figure 1i). Besides, x‐ray diffraction (XRD) pattern only shows the diffraction peaks indexed to the Cr_2_O_3_, indicating the low content and uniform distribution of Au NPs (Figure S5b). Thermogravimetric analysis (TGA) illustrated that the Au@mCr_2_O_3_‐1.0 retains approximately 85% of its mass at 800°C, indicating a thermally stable framework. The thermal stability of the Au@mCr_2_O_3_ matrix ensure that the absorbed laser energy is primarily utilized for efficient desorption/ionization of analytes rather than matrix degradation, thereby guaranteeing highly reproducible and reliable metabolic fingerprints. (Figure S6).

### 2.2 Exploration of Au@mCr_2_O_3_‐based MALDI‐MS performance

Owing to the optimized topological structure and the tailored chemical microenvironment, Au@mCr_2_O_3_ provides enhanced signal intensity for MALDI‐MS detection, thereby facilitating the identification of low‐abundant metabolites. During the detection of standard small‐molecule metabolites, Au@mCr_2_O_3_‐1.0 exhibits the highest typical peak intensities toward taurine (Tau), valine (Val), glutamic (Glu), Glucose (Glc), and aspartic acid (Asp), with 1.6–6‐fold enhancement compared to that of mCr_2_O_3_ (Figure 2a; Figure S7). The enhancement in signal intensity can be attributed to the improvement in ionization and desorption efficiency, which depends on the photogenerated carriers yield and photothermal conversion efficiency of matrix material. Notably, UV–vis absorption spectra reveal that the mCr_2_O_3_ exhibits extended absorption at 355 nm after the decoration of Au NPs, matching well with the laser wavelength of MALDI‐MS (Figure S8). Moreover, XPS analysis indicates that the introduction of Au NPs increases the oxygen vacancies (O_v_) contents in the matrix material (Figure 2b; Figure S9), which can promote the electron transfer and the local energy confinement. Therefore, the enhanced broad‐band light harvesting and the increased O_v_ concentration are expected to synergistically improve the ionization and desorption efficiency of MALDI‐MS. Furthermore, electron paramagnetic resonance (EPR) data confirmed the abundant presence of these bulk oxygen vacancies by providing the characteristic signal of unpaired electrons trapped at the oxygen vacancies, with the maximum intensity observed for the Au@mCr_2_O_3_‐1.0 (Figure S10).

**FIGURE 2:**
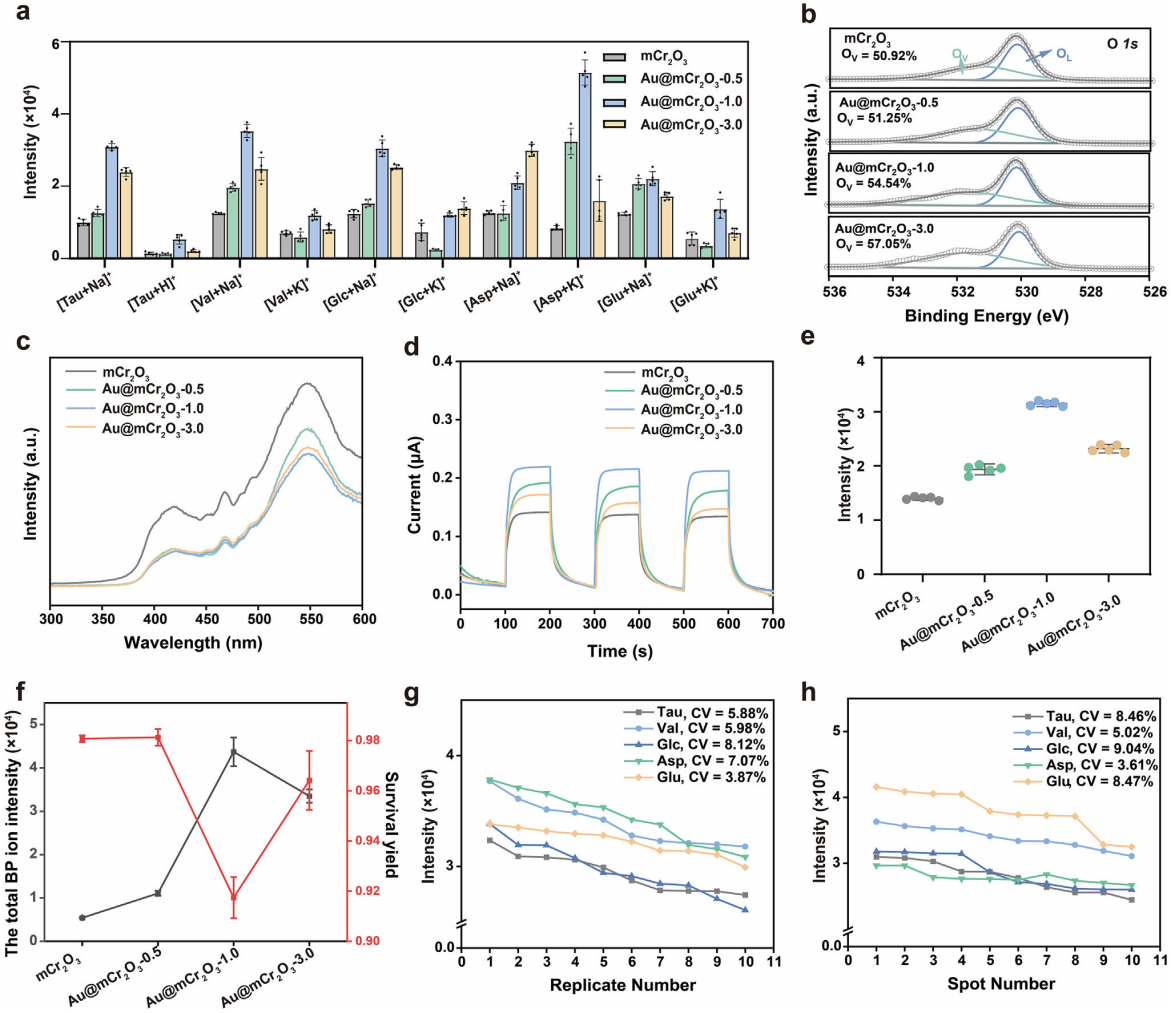
Exploration of Au@mCr_2_O_3_‐based MALDI‐MS performance. (a) MALDI‐MS performance of mCr_2_O_3_ and Au@mCr_2_O_3_‐n as matrices for detecting standard metabolites. (b–d) O 1*s* XPS spectrum (b), PL spectra (c), and photocurrent analysis (d) of mCr_2_O_3_ and Au@mCr_2_O_3_‐n. (e) Peak intensity of indigo using mCr_2_O_3_ and Au@mCr_2_O_3_‐n as matrices. (f) The total intensity of BP ions (black line) and survival yield (SY) of parent ions (red line) desorbed from mCr_2_O_3_ and Au@mCr_2_O_3_‐n. (g) Coefficients of variation of MALDI‐MS signals from the standard metabolites using Au@mCr_2_O_3_‐1.0 as matrices, replicating 10 times independent experiments on the same spot. (h) Coefficients of variation of MALDI‐MS signals from the standard metabolites using Au@mCr_2_O_3_‐1.0 as matrices, replicating 10 times independent experiments on different spots. Quantitative data in a, e, and f are presented as mean ± standard deviation (SD) derived from five independent replicates.

To further elucidate the underlying mechanism, we systematically investigated the photogenerated carrier dynamics and the photothermal conversion capability of Au@mCr_2_O_3_. Photoluminescence (PL) emission spectroscopy and photocurrent measurement were employed to evaluate the efficiency of charge separation. Compared with mCr_2_O_3_, Au@mCr_2_O_3_‐n particles exhibit lower PL intensities and higher photocurrent response due to the electron‐trapping effect of Au NPs that suppress the electron‐hole recombination (Figure 2c,d). The low recombination rate of photogenerated carriers is conductive to charge transfer, thereby facilitating the ionization of analytes. Indigo is further employed as a molecular probe to directly assess the ionization efficiency of matrix owing to its chemical stability. Since the signal intensity is positively correlated with the ionization efficiency, the enhancement of the indigo signal intensity after Au NPs decoration indicates the improvement of the ionization efficiency, and the signal intensity increases with the rising of laser energy (Figure 2e; Figures S11 and S12). Among the Au@mCr_2_O_3_‐n matrices, Au@mCr_2_O_3_‐1.0 displays the highest ionization efficiency.

In addition to photogenerated carrier dynamics, the photothermal conversion capability of matrix materials is also explored. Under laser irradiation, Au NPs generate localized surface plasmon resonance to produce hot carriers. The hot carriers rapidly dissipate into heat, thereby achieving thermal‐driven desorption of analytes. Monitoring the temperature of the matrix suspensions under laser irradiation reveals that an increase in the decoration amount of the Au NPs would lead to the elevation in temperature (Figure S13). Benzyl pyridinium (BP) is used as a chemical thermometer to assess the photothermal conversion performance of Au@mCr_2_O_3_. The total intensity of BP, including parent BP ion and its fragmentation [BP‐pyridine]^+^, is positively correlated with the desorption efficiency of analytes and reflects the photothermal conversion capability of matrix. In contrast, survival yield (SY) is negatively correlated with the efficiency of thermal‐driven desorption. Generally, Au@mCr_2_O_3_ matrices show higher total intensities and lower SY than mCr_2_O_3_, and Au@mCr_2_O_3_‐1.0 achieves the most efficient desorption performance among the Au@mCr_2_O_3_‐n matrices (Figure 2f; Figure S14).

Owing to the outstanding photogenerated carrier dynamics and photothermal conversion capability, Au@mCr_2_O_3_‐1.0 is considered as the optimal candidate of matrix material. Furthermore, the performance of Au@mCr_2_O_3_‐1.0, including sensitivity, reproducibility, and selectivity, were comprehensively investigated. The sensitivity was evaluated by detecting the limit of detection (LoD) of representative small‐molecule metabolites, including Val (10 pmol), Glu (50 pmol), Glc (25 pmol), histidine (His, 50 pmol), and aspartic acid (25 pmol) (Table S1). These values are significantly lower than the endogenous concentrations, exhibiting the potential of Au@mCr_2_O_3_‐1.0 as matrix for detecting low‐abundance small molecules. Considering that the abundance and complexity of the biological fluid sample would interfere with MS signals, a complex environment was established by incorporating Glu, His, Glc, Asp, L‐phenylalanine (Phen), Val, Aspartic acid (Asp), and Tau, and all metabolites can be successfully detected by the Au@mCr_2_O_3_‐1.0 (Figure S15a, Table S2). Moreover, the Au@mCr_2_O_3_‐1.0 is capable of quantitative detecting the concentrations of metabolites. For instance, the signal intensities of Glu and Val show good linear relationship with their concentrations, with correlations coefficients (R^2^) of 0.9872 and 0.9974, respectively (Figure S16). Owing to the poor‐crystallization ability with analytes and weak stability under laser irradiation, conventional matrices such as 2,5‐dihydroxybenzoic acid (DHB) and α‐cyano‐4‐hydroxycinnamic acid (CHCA) usually exhibit high coefficients of variation (CV) value (17.63%–86.45%) and obvious background noise in the low‐mass region (Figures S17–S19). By contrast, the CV value of Au@mCr_2_O_3_‐1.0 during the repeated measurements at different positions within the same spot and across different spots range from 3.61% to 9.04%, indicating the good stability of this mesoporous semiconductor‐based detection platform (Figure 2g,h). Moreover, even under high‐salt (NaCl, 0.5 M) and high‐protein (bovine serum albumin (BSA) solution, 5 mg mL^−1^) conditions, Au@mCr_2_O_3_‐1.0 still demonstrates good environmental tolerance, which is conductive to its practical applications in biological fluid analysis (Figure S15b, c). Based on its outstanding performance, Au@mCr_2_O_3_‐1.0 is conducted as the matrix to establish a detection platform for the following investigation.

Compared to commercial inorganic‐matrix TiO_2_ nanoparticles (P25) and disordered mesoporous Au@Cr_2_O_3_ (D‐Au@mCr_2_O_3_‐1.0), Au@mCr_2_O_3_‐1.0 achieved significantly enhanced signal intensities for both standard metabolites and the tetrabutylammonium (TBA^+^) probe (Figure S20a, b). Furthermore, BP^+^ thermometer analysis confirmed that its continuous mesoporous framework provides the most efficient photothermal energy transfer for optimal analyte desorption (Figure S20c–e). Therefore, Au@mCr_2_O_3_‐1.0 exhibited the highest signal intensity for standard metabolite detection (Figure S20f).

To maximize the performance of the Au@mCr_2_O_3_‐1.0 matrix, the matrix‐to‐analyte ratio was systematically investigated. The highest signal intensity was observed at a matrix concentration of 1.0 mg mL^−1^, which can be attributed to the highly uniform co‐crystallization and excellent desorption/ionization efficiency (Figure S21). Therefore, 1.0 mg mL^−1^ was selected as the optimal matrix concentration for all subsequent experiments. To further evaluate the broader applicability of Au@mCr_2_O_3_‐based MALDI‐MS detection platform, metabolic profiles were successfully acquired from human plasma, urine, and saliva samples using the standardized protocol (Figure S22). These results strongly demonstrate the versatility of the platform across various biofluids and verify the translational potential of the platform for disease diagnosis in diverse clinical scenarios.

### 2.3 Establishment of Saliva Metabolic Fingerprint for Periodontitis Diagnosis

Salivary metabolites can non‐invasively reflect the local periodontal inflammation status with high sensitivity, making them an ideal bio‐sample for the early screening of periodontitis. Fingerprint profiles of saliva metabolites of periodontitis disease (PD) were analyzed using the Au@mCr_2_O_3_‐based MALDI‐MS detection platform, and a classification model was constructed through machine learning. In this study, 191 saliva samples were collected, including 59 healthy controls (HC) and 132 PD samples. All PD patients were diagnosed through clinical examination and imaging results. Detailed information about these samples is presented in Figure 3a. There is no statistically significant difference in gender distribution between the HC and the PD group by *χ^2^* test (Table S3). Epidemiological studies indicate that PD increases with age, affecting approximately 40% of adults worldwide and becoming more common among individuals over 35. The age distribution of the PD group is consistent with the typical trend of an increasing prevalence of periodontitis among middle‐aged and elderly people. By using the Au@mCr_2_O_3_‐based detection platform to record representative mass spectra of saliva metabolites in the low molecular weight range (100–500 Da), significant differences in metabolic peaks between the HC and the PD can be observed (Figure 3b). In parallel, minimal within‐group variation in cosine similarity indicates the high stability of data (Figure 3c). After processing the original data with RStudio, 443 *m/z* features were extracted, and a robust SMFs was established for subsequent analysis. Heatmap illustrates subtle variations in patterns within the SMFs, underscoring the necessity for a highly sensitive and reliable analysis strategy (Figure 3d). Subsequently, principal component analysis (PCA) was applied as an unsupervised method, but it failed to achieve a clear separation between the HC and the PC groups (Figure 4a).

**FIGURE 3:**
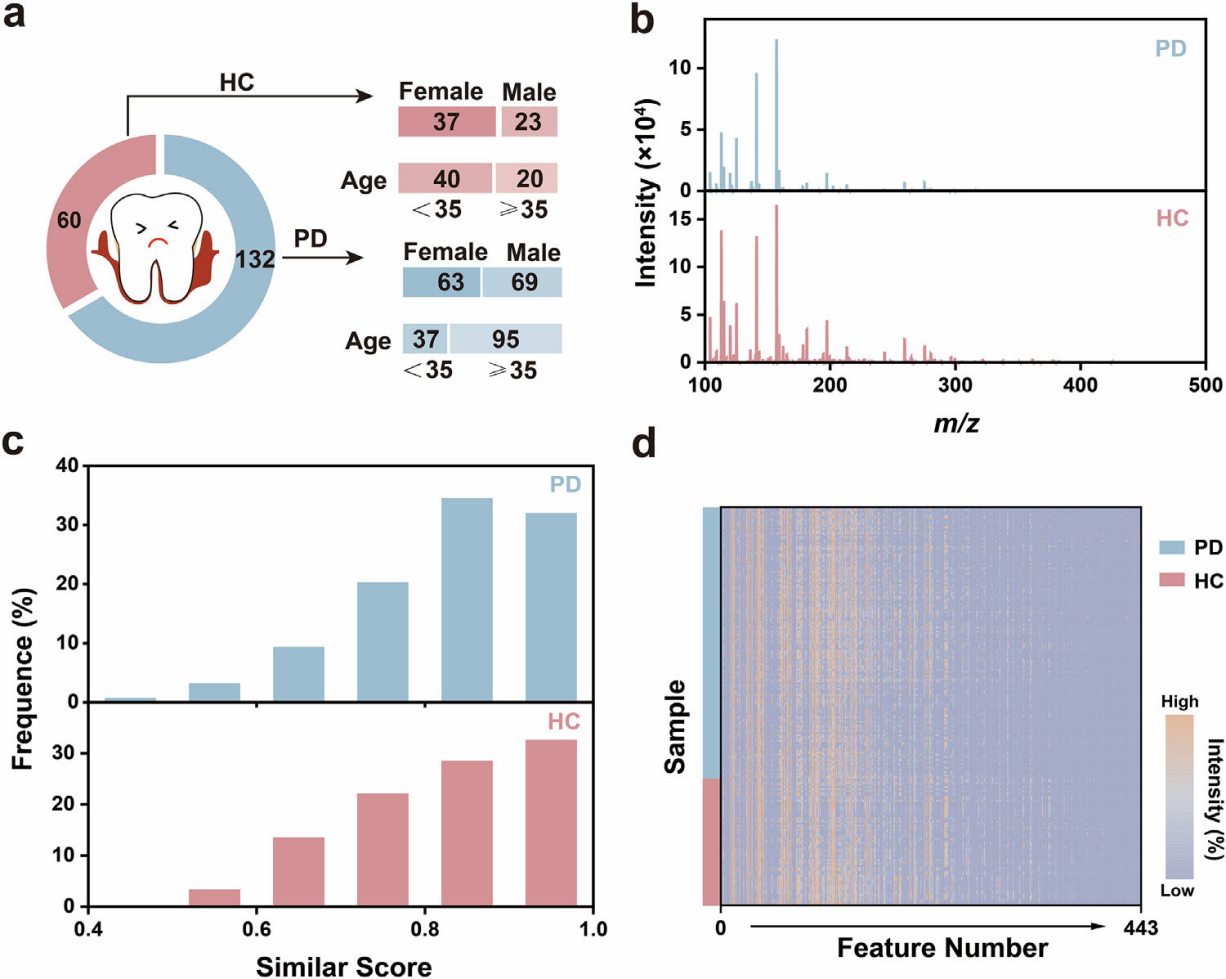
Extraction of the SMFs. (a) Age and gender distribution of the HC and PD groups. (b) Typical saliva MS spectra of one HC and one PD in the m/z range of 100–500 Da. (c) Distribution of similarity scores of saliva metabolic fingerprints of the HC and PD groups. (d) Heatmap of all features extracted from data processing.

**FIGURE 4:**
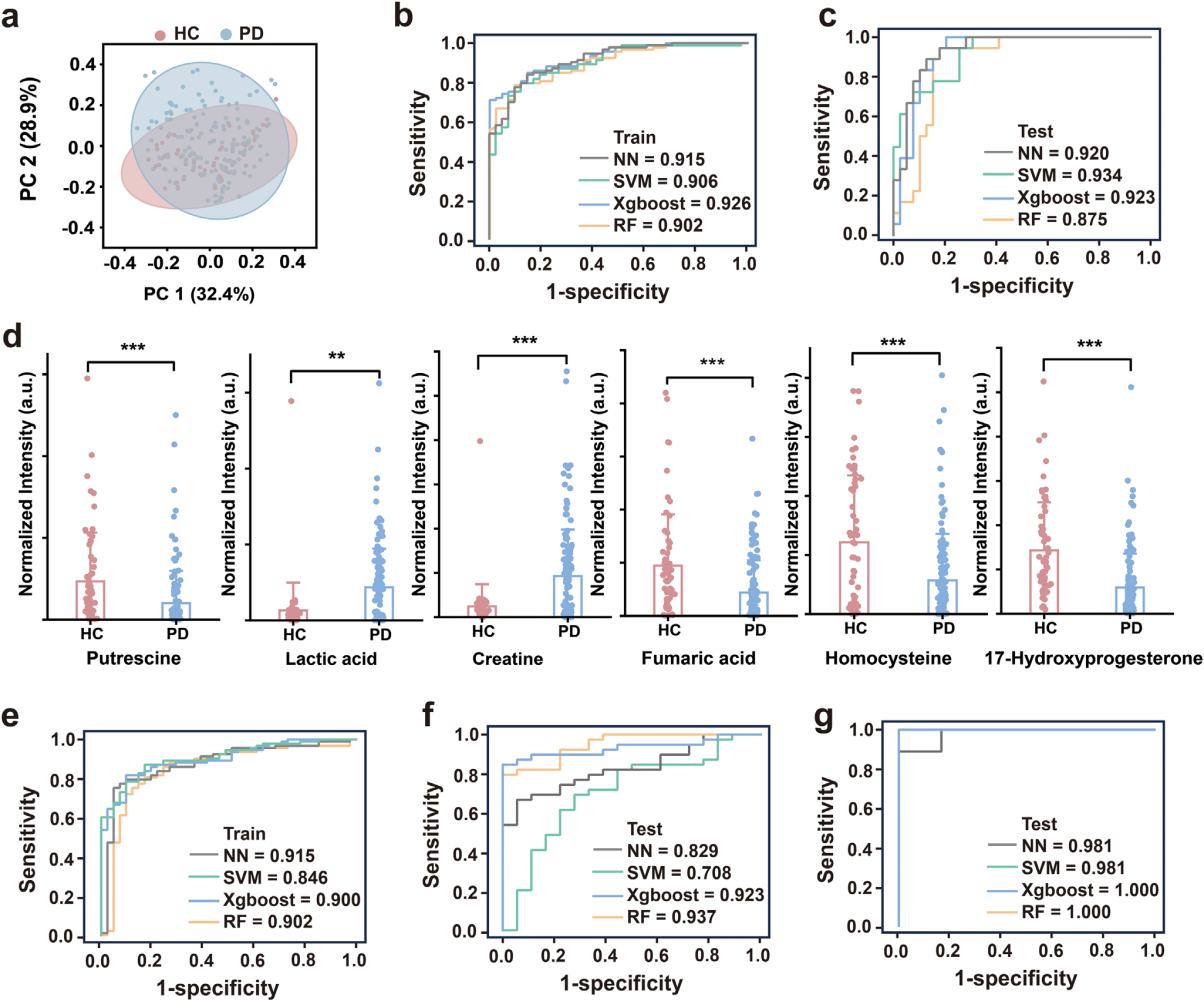
Diagnosis of periodontitis based on SMFs. (a) PCA scores plot of PD and HC. (b, c) The receiver operating characteristic (ROC) curves based on SMFs in the train cohort (b) and test cohort (c) for four machine learning including NN, SVM, Xgboost, and RF. (d) Normalized intensities of six biomarkers in the HC and PD group (^**^ *p* < 0.01, ^***^ *p* < 0.001). (e, f) ROC curves by four machine learning algorithms based on biomarkers in the train cohort (e) and test cohort (f). (g) ROC curves by four machine learning algorithms based on biomarkers in the blind test cohort.

Because it can extract key features from high‐dimensional and complex data to enhance the discriminative ability of the model, machine learning was introduced to decode the complex SMFs of PD. All samples were randomly divided into a train set (HC/PD, 41/93) and a test set (HC/PD, 18/39) at the ratio of 7:3. Four machine learning algorithms, including neural network (NN), extreme gradient boosting (Xgboost), support vector machines (SVM), and random forest (RF), were employed to classify PD in these datasets. To comprehensively evaluate the performance of these models, area under the curve (AUC), accuracy, precision, recall, and F1 scores were calculated. All algorithms achieve AUC ≥ 0.85, demonstrating the strong potential of this platform in accurately identifying PD (Figure 4b,c). Meanwhile, Xgboost shows the best performance with an AUC of 0.926 (95% confidence interval (CI): 0.868–0.984), accuracy of 0.821, F1 score of 0.817, recall of 0.821, and precision of 0.816 in the train set (Figure 4b; Table S4), which are higher than that obtained by SVM (AUC of 0.906, accuracy of 0.828, F1 score of 0.829, recall of 0.828, and precision of 0.830), NN (AUC of 0.915, accuracy of 0.828, F1 score of 0.831, recall of 0.828, and precision of 0.836) and RF (AUC of 0.902, accuracy of 0.806, F1 score of 0.803, recall of 0.806, and precision of 0.802). In the test set, the Xgboost algorithm also maintains excellent performance, with an AUC of 0.923 (95% CI: 0.834–1.000) (Figure 4c; Table S4). Furthermore, sample prediction distribution plots based on all four algorithms show a significant separation between the HC and the PD groups, further confirming the robustness and potential of this platform for accurate PD diagnosis (Figures S23 and S24).

### 2.4 Construction of a Simplified PD‐Recognizer

The simplified PD‐recognizer provides a convenient and efficient identification for practical diagnosis applications. The key to constructing a simplified PD‐recognizer lies in establishing a reliable biomarker panel that directly reflects the biochemical activity within the microenvironment of the organism. Based on RelfiefF (top 80 features) and filter methods (*p*<0.05, fold change [FC] > 2 or<0.5), 24 *m/z* features were initially screened (Figure S25). Among them, six metabolite features were identified through matching with the Human Metabolome Database (HMBD, http://www.hmdb.ca/), including putrescine, lactic acid, creatine, fumaric acid, homocysteine, and 17‐hydroxyprogesterone (Figure S26, Table S5). These biomarkers exhibit physiological‐level variation associated with different health states. Specifically, compared to the HC group, the putrescine, fumaric acid, homocysteine, and 17‐hydroxyprogesterone in the PD group are down‐regulated (*p*<0.05), while the lactic acid and creatine are up‐regulated (*p*<0.05) (Figure 4d).

Afterward, the simplified PD‐recognizer was constructed based on the biomarker panel, displaying satisfactory classification performance. All machine learning algorithms achieved AUC values above 0.7, indicating the robustness and reliability of the biomarkers panel for PD‐recognizer (Table S6). The Xgboost algorithm consistently demonstrated high classification accuracy with an AUC of 0.900 (95% CI: 0.834–0.966) in the train set and 0.933 (95% CI: 0.849–1.000) in the test set (Figure 4e,f). The predicted probability distribution plots further indicates that there is a significant separation between the HC and the PD groups (Figures S27 and S28). To preliminarily evaluate the practical applicability of the simplified PD‐recognizer, we recruited an additional independent saliva cohort consisting 15 samples (HC/PD, 6/9) as a blind external validation cohort. Despite the limited cohort size, all machine learning models achieve AUC values of 0.981–1.000 in this independent cohort. Among them, Xgboost, NN, and RF only made one incorrect classification for the samples, demonstrating the promising potential of this platform in accurate and non‐invasive PD recognition (Figure 4g; Figures S29 and S30).

The non‐invasive diagnosis model tracks dynamic metabolic changes to achieve real‐time evaluation of periodontal health, allowing for timely intervention to prevent tooth loss. To gain a deeper understanding of metabolic mechanisms of periodontitis and verify the biological reliability of the targeted biomarker panel, Metaboanalyst 6.0 (https://www.metaboanalyst.ca/) was used to analyze the metabolic pathway of the identified biomarkers. As shown in the Figure S31, the six identified biomarkers are primarily involved in seven metabolic pathways, containing (1) arginine and proline metabolism, (2) cysteine and methionine metabolism, (3) citrate cycle (TCA cycle), (4) steroid hormone biosynthesis, (5) tyrosine metabolism, (6) glutathione metabolism, and (7) alanine, aspartate and glutamate metabolism (Table S7). Based on these findings, it is concluded that the metabolites mainly participate in energy metabolism remodeling and inflammatory response regulation during the periodontitis progression. Specifically, lactic acid, creatine, and fumaric acid are associated with energy metabolism, whereas putrescine, homocysteine, fumaric acid, and 17‐hydroxyprogesterone are associated with inflammation/immune regulation [42]. Given that periodontitis is one of the most prevalent chronic inflammatory diseases, the altered metabolic pathways identified here are closely related to inflammatory processes [33, 34, 35, 36, 37, 38, 39, 40, 41, 42, 43, 44, 45, 46, 47]. Overall, the biomarker panel offers mechanistic insights into the pathophysiology of periodontitis and is expected to improve the disease diagnosis and guide the development of targeted therapeutic strategies.

Although the diagnostic performance achieved in this work is promising, certain limitations must be acknowledged. The primary purpose of this work is to present a proof‐of‐concept platform for periodontitis detection. The blind test cohort provides initial external validation, but the sample size remains relatively limited. Future large‐scale clinical cohort validations are essential to comprehensively assess the applicability of this biomarker panel across broader populations.

## 3 Conclusion

A non‐invasive periodontitis detection platform was established by MALDI‐MS based on mesoporous Au@mCr_2_O_3_ particles as matrix. The mCr_2_O_3_ particles with single primitive continuous architecture and Schwarz P surface were rationally designed and synthesized through the PCs‐templating method, and Au NPs as co‐catalysts were further introduced on the pore wall to promote the photogenerated carrier dynamics and photothermal conversion capability. Due to the optimized nanoarchitecture and customized chemical microenvironment, the Au@mCr_2_O_3_‐based MALDI‐MS exhibits enhanced ionization and desorption efficiency, enabling the sensitive detection of metabolite and the successful establishment of SMFs. Through machine learning and multivariate statistical analysis of the SMFs, a metabolic biomarker panel, including putrescine, lactic acid, creatine, fumaric acid, homocysteine, and 17‐hydroxyprogesterone, was identified and used to construct a periodontitis diagnostic model. The machine learning model achieved favorable classification of periodontitis with an AUC of 0.981–1.000 in the blind test sets. This work presents a proof‐of‐concept platform for periodontitis detection, which achieved user‐friendly, rapid, and accurate discrimination of periodontitis. Furthermore, this study not only inspire the rational design of advanced matrix materials and offers a reliable detection platform for diseases diagnosis, but also deepens our understanding of the disease‐associated metabolic signatures, which may facilitate future studies on disease mechanisms and translational application.

## 4 Experimental Section

### 4.1 Chemicals and Materials

Methoxypolyethylene glycol (PEO, M_w_ = 2000 g/mol), copper(I) bromide (CuBr), tetrahydrofuran (THF), 2‐bromoisobutyryl bromide (C_4_H_6_Br_2_O, 98%), N, N, N′, N″, N″‐pentamethyldiethylenetriamine (PMDETA, 99%), styrene (St), 1,4‐Dioxane, N, N‐dimethylformamide (DMF), acetonitrile (ACN), and gold chloride trihydrate (HAuCl_4_·3H_2_O) were purchased from Aladdin. 2,5‐dihydroxybenzoic acid (DHB) and 𝛼‐cyano‐4‐hydroxycinnamic acid (CHCA) were bought from Adamas‐beta. L‐valine (Val), L‐histidine (His), L‐phenylalanine (Phen), D‐glucose (Glc), L‐aspartic acid (Asp), D‐glutamic acid (Glu), L‐arginine (Arg), Taurine (Tau), and bovine serum albumin (BSA) were purchased from Sigma–Aldrich. chromium (III) nitrate nonahydrate (Cr(NO_3_)_3_·9H_2_O, ≥99%) was purchased from Sinopharm Chemical Reagent Co.

### 4.2 Characterizations and Measurements

High‐resolution TEM images and high‐angle annular dark‐field scanning transmission electron microscopy (HAADF‐STEM) images were obtained at a Talos F200X G2 field emission transmission electron microscopy (FEI, America) at 200 kV. XPS was carried out on an AXIS ULTRA DLD XPS System with MONOAl source (Shimadzu Corp., Japan). XRD patterns were collected at a Bruker D4 X‐ray diffractometer (Germany) equipped with Ni‐filtered Cu Kα radiation (40 kV, 40 mA) and dealt with Jade 6 (Jade Software Corporation Ltd.). UV–vis absorption spectra were collected on Lambda 1050+ (PE, America). Matrix‐assisted laser desorption/ionization mass spectrometry (MALDI‐MS) analyses were performed using a Bruker Autoflex Speed MALDI‐TOF/TOF mass spectrometer (Bruker Daltonics, ultrafleXtreme, Germany) equipped with a 355 nm Nd:YAG solid‐state laser. Data acquisition was conducted in positive ion reflection mode with a mass range of m/z 100–1000 and a laser repetition rate of 2000 Hz. The photoluminescence (PL) spectra of the samples were recorded using a fluorescence spectrometer (FLS1000, Edinburgh Instruments, UK). N_2_ adsorption‐desorption isotherms were obtained at 77 K with an ASAP 2420 analyzer (ASAP 2020 Plus HD88, America). The specific surface area data were recorded with the Brunauer‐Emmett‐Teller (BET) method, and the size distributions were dealt with the Barrett‐Joyner‐Halenda (BJH) model.

### 4.3 Synthesis of Polymer Cubosomes

PEO‐*b*‐PS copolymers with a molecular composition of PEO_45_‐*b*‐PS_278_ and polydisperse index of 1.11 were prepared by atom‐transfer radical polymerization (ATRP). 50 mg of PEO_45_‐*b*‐PS_278_ was dissolved in dioxane/DMF (v/v, 46:4). Then, 1 mL of deionized water was gradually added during 1 h to induce the formation of polymer cubosomes. The formed suspension was stirred gently for 3 h, followed by quickly pouring into 20 mL of deionized water to freeze the morphology. Finally, the polymer cubosomes were collected by centrifugation and washed with deionized water.

### 4.4 Synthesis of mCr_2_O_3_

0.2 mL of Cr(NO_3_)_3_·9H_2_O (0.5 g/mL) aqueous solution was added to the polymer cubosomes assembled by 50 mg of PEO‐*b*‐PS. Dry the mixture under vacuum conditions at 45°C. The obtained powders were calcined in tube furnace at 450°C for 1 h under a N_2_ atmosphere (heating rate of 1°C/min to 350°C, and then 5°C/min to 450°C). Finally, the powders were calcined in muffle furnace at 400°C for 0.5 h under an air atmosphere (heating rate of 5°C/min) to obtain mCr_2_O_3_ particles.

### 4.5 Synthesis of Au@mCr_2_O_3_‐n (n = 0.5, 1.0, and 3.0) Particles

Disperse 20 mg of the mCr_2_O_3_ in 5 mL of ethanol. Then, add the corresponding amounts of HAuCl_4_ aqueous solution (5 mg/mL, 0.08 mL for 1.0 wt.% Au loading) into the mCr_2_O_3_ suspension, and stir the mixture at 350 r/min for 2 h. Then, the mixture solution was transferred to a drying oven to evaporate the solvent at 70°C. Finally, the obtained power was calcined at 350°C for 2 h under a H_2_ atmosphere (heating rate of 5°C/min). By controlling the feeding ratio of HAuCl_4_, mCr_2_O_3_ particles with different decoration amount of Au NPs were obtained (Au@mCr_2_O_3_‐n, n = 0.5, 1.0, and 3.0).

### 4.6 Synthesis of D‐Au@mCr_2_O_3_‐1.0

20 mg of PEO‐*b*‐PS was dissolved in 5 mL THF by ultrasonic to form solution A, and 500 mg of Cr(NO_3_)_3_·9H_2_O was dissolved in a mixture of 4 mL of ethanol and 40 µL of HCl and HAc by ultrasonic to form solution B. Then, solution A and B were mixed under stirring for 2 h. The mix solution was poured into Petri dishes to evaporate solvent in a hood at 25°C for 24 h. The composites on the glass substrate of Petri dishes were further heated at 40°C in an oven for 12 h to completely remove solvent and annealed at 100°C for another 12 h to form transparent film. The film was scraped off and crushed into powder which was thermally treated at 450°C for 1 h under a N_2_ atmosphere (heating rate of 1°C/min to 350°C, and then 5°C/min to 450°C). Then, the powders were calcined in muffle furnace at 400°C for 0.5 h under an air atmosphere (heating rate of 5°C/min) to obtain D‐mCr_2_O_3_ particles. Disperse 20 mg of the D‐mCr_2_O_3_ in 5 mL of ethanol. Then, add the corresponding amounts of HAuCl_4_ aqueous solution into the D‐mCr_2_O_3_ suspension, and stir the mixture at 350 r/min for 2 h. Then, the mixture solution was transferred to a drying oven to evaporate the solvent at 70°C. Finally, the obtained power was calcined at 350°C for 2 h under a H_2_ atmosphere (heating rate of 5°C/min). By controlling the feeding ratio of HAuCl_4_, D‐Au@mCr_2_O_3_‐1.0 particles were obtained.

### 4.7 Synthesis of Thermometer Molecules

The benzyl pyridinium (BP) was used as the chemical thermometer to evaluate the ion desorption efficiency. BP was synthesized according to the reported work. 2 mL of pyridine and 0.1 of mL benzyl chloride were added to a 50 mL flask and stirred at 60°C for 5 h. Subsequently, the reaction mixture was subjected to rotary evaporation to remove the excess pyridine. The products were dissolved in the MeOH and H_2_O mixture solution (MeOH: H_2_O, v/v = 1:1) to obtain the 0.1 mM [BP]^+^ solution.

$${\left[\mathrm{BP}\right]}^{+}\to {\left[\mathrm{BP}-\mathrm{pyridine}\right]}^{+}+\mathrm{pyridine}$$

$$\mathrm{SY}\;=\left[I\left(m/z\;170\right)]/([I\left(m/z\;170\right)]+[\left(I\left(m/z\;91\right)\right]\right)$$

### 4.8 Collection of Saliva Samples

All saliva samples were collected according to the standard clinical protocols [48]. Periodontitis is primarily diagnosed based on clinical and imaging examinations, in accordance with the 2018 International Classification of Periodontitis. Crucially, to eliminate long‐term confounding factors (e.g., systemic diseases), strict inclusion and exclusion criteria were applied during recruitment, and participants with major systemic diseases were excluded. To minimize short‐term interferences such as smoking and diet, unstimulated whole saliva was collected under standardized and supervised conditions, requiring participants to refrain from eating, drinking, smoking, or performing oral hygiene for at least 2 h prior to sample collection. The collected samples were immediately placed at 4°C. Subsequently, the samples were centrifuged at 3500 rpm for 10 min to collect the liquid supernatant, which was then aliquoted and stored at −80°C before use. The study was approved by the Ethics Committee of Shanghai Stomatological Hospital, Fudan University (No.20240017), and registered in the P. R. China Clinical Trial Registry (No. ChiCTR2500102618).

### 4.9 MALDI‐MS Detection

To detect standard metabolites, all standard metabolites were dissolved in deionized water. For the mixture solution of standard metabolites, 20 µL of each metabolite solution (Glu, His, Glc, Asp, Phe, Val, Arg, and Tau at 20 mM) was mixed. To evaluate the selectivity of matrix materials, high‐salt and protein‐containing solutions were prepared by adding NaCl (0.5 m) and BSA (10 mg/mL) to the mixture solution. As the organic matrices, CHCA (10 mg/mL) and DHB (10 mg/mL), were dissolved in a 0.1% TFA solution (deionized water: ACN = 1:1, v/v). As the matrix solution, mCr_2_O_3_ and Au@mCr_2_O_3_‐n (n = 0.5, 1.0, 3.0) particles were dissolved in deionized water with a concentration of 1.0 mg/mL to obtain the matrix solution. The sample preparation process is as follows: 1 µL of analyte solution and 1 µL of matrix suspension solution were sequentially dropped on the target plate and dried at room temperature for detection. Mass spectra analysis was collected using UlrafleXtreme MALDI‐TOF/TOF (Bruker) system equipped with a 355 nm Nd: YAG laser in positive ion reflector mode, operating at a repetition rate of 2000 Hz and an acceleration voltage of 20 kV. The accelerating voltage was 20 kV, and the intensity of the laser was 80%.

### 4.10 The Working Principle of MALDI‐MS

Initially, the trace analyte and a large excess of matrix molecules are deposited onto the target plate to form co‐crystals. Following exposure to short laser pulses, the photon energy is intensely captured by the matrix, triggering an explosive desorption. This sudden vaporization carries the undamaged analyte molecules into a gaseous plume, where they achieve ionization through charge‐exchange mechanisms. Ultimately, these newly charged species are propelled toward a mass spectrometer to record their *m/z* values.

### 4.11 Data Analysis

All original mass spectra data were obtained using Bruker FlexAnalysis (version 3.4). The MALDIquant package was used for averaging and normalizing the mass spectra of bio‐samples in RStudio. Machine learning algorithms, including support vector machine (SVM), neural network (NN), random forest (RF), and extreme gradient boosting (Xgboost) were performed using the Orange software (version 3.38.1. A 10‐fold stratified cross‐validation strategy was employed during model training to evaluate performance and mitigate overfitting. The PCA model and pathway analysis were performed on the MetaboAnalyst 5.0 (https://www.metaboanalyst.ca/). Similarity scores were acquired by Python. The biomarker metabolites were identified on the Human Metabolome Database (HMDB, http://www.hmdb.ca/).

### 4.12 Statistical Analysis

Statistical analyses were performed using GraphPad Prism (GraphPad Software, Inc., San Diego, CA). To address the false positive risk associated with multiple hypothesis testing in high‐dimensional metabolomics data, the original *p*‐values obtained from univariate analyses were adjusted using the Benjamini‐Hochberg False Discovery Rate (FDR) procedure. Features with an FDR‐adjusted *p* < 0.05 were considered statistically significant. The probability value (*p*‐value) < 0.05 was considered statistically significant. Statistical significance was set at ^*^ *p* < 0.05, ^**^ *p* < 0.01, ^***^ *p* < 0.001).

## Author Contributions

**Yan Wang**: data curation, investigation, formal analysis, validation. **Wenhe Xie**: writing – review and editing, supervision, methodology. **Limin Wu**: supervision, project administration, writing – review and editing. **Chunhui Deng**: methodology. **Yiding Zhang**: data curation. **Heyuhan Zhang**: formal analysis. **Meihua Chen**: writing – review and editing, data curation, methodology, conceptualization. **Fangying Shi**: formal analysis, methodology, validation. **Yonghui Deng**: conceptualization, supervision, project administration, writing – review and editing, investigation, formal analysis. **Yue Sun**: conceptualization, data curation, formal analysis, visualization, writing – original draft, methodology, investigation, validation.

## Conflicts of Interest

The authors declare no conflict of interest.

## Supporting information




**Supporting File**: advs76441‐sup‐0001‐SuppMat.docx.

## Data Availability

The data that support the findings of this study are available in the supplementary material of this article.
