# Supplementary material for: An Efficient Detection Platform Based on Mesoporous Au@Cr2O3 Particles with Schwarz P Surface for Precise Periodontitis Metabolite Profiling
Source: Adv Sci (Weinh). 2026 Jul 27;13(54):e76441. doi: 10.1002/advs.76441 (PMC13403884; doi:10.1002/advs.76441)
Supplement: Supplementary file 1 [file ADVS-13-e76441-s001.docx]

Supporting Information

An Efficient Detection Platform Based on Mesoporous Au@Cr_2_O_3_ Particles with Schwarz P Surface for Precise Periodontitis Metabolite Profiling

Yue Sun^[a]^, Yan Wang^[a]^, Fangying Shi^[b]^, Wenhe Xie*^[a]^, Yiding Zhang^[a]^, Heyuhan Zhang^[a]^, Limin Wu*^[c]^, Meihua Chen*^[a]^, Yonghui Deng*^[a]^

Figure S1. The structural analysis of PCs. a, b) SEM images of PCs (a) and the region of PCs (b). PCs shows spherical morphology with one set of pore channels is opened pore (blue area) and the other set is sealed pore channel (red area). The pores are uniformly distributed on the surface of PCs in a square lattice belonging to double primitive topology. Lt means the length of lattice parameter. c) SAXS pattern of PCs (lattice parameter $\boldsymbol{a=}\boldsymbol{2\pi}/{\boldsymbol{q}^{\boldsymbol{*}}}$).). SAXS pattern exhibits characteristic peaks with a *q* ratio of $\sqrt{\boldsymbol{2}}\boldsymbol{:}\sqrt{\boldsymbol{4}}\boldsymbol{:}\sqrt{\boldsymbol{6}}\boldsymbol{:}\sqrt{\boldsymbol{8}}$, belonging to double primitive cubic structure (*Im*$\bar{\boldsymbol{3}}\boldsymbol{m}$ symmetry).

Figure S2. The structure of mCr_2_O_3_ particles. a, b) SEM images of mCr_2_O_3_ particles (a) and the region of mCr_2_O_3_ particles (b). The surface of mCr_2_O_3_ particles shows uniform pore distribution in a square lattice manner, attributing a single primitive topology.

**Figure S3.** a, b) Nitrogen adsorption-desorption isotherm (a) and pore size distribution (b) of mCr_2_O_3_ particles. The specific surface area is 75.2 m^2^/g, and the average mesopore size is about 39 nm.

**Figure S4.** XPS spectra of mCr_2_O_3_ particles. a) Cr *2p* spectrum shows two peaks attributed to the spin-orbitals of Cr *2p_3/2_* and Cr *2p_1/2_*. b) O *1s* spectrum with peaks at 530.1 and 531.2 eV, which belong to lattice oxygen (O_L_) and oxygen vacancies (O_V_), respectively.

Figure S5. a) SAXS pattern of Au@mCr_2_O_3_-1.0 (lattice parameter $\boldsymbol{a=}\boldsymbol{2\pi}/{\boldsymbol{q}^{\boldsymbol{*}}}$). b) XRD patterns of mCr_2_O_3_ and Au@mCr_2_O_3_-n (n = 0.5, 1.0, 3.0).

Figure S6. TG curves of Au@Cr_2_O_3_-1.0.

Figure S7. a–d) The typical mass spectrum of mCr_2_O_3_ and Au@mCr_2_O_3_-n (n = 0.5, 1.0, 3.0) particles as matrix for detecting Tau (a), Asp (b), Glc (c), and Val (d). The peaks of [M + Na]^+^ and [M + K]^+^ are labeled (M represents Tau, Asp, Glc, and Val, respectively).

Figure S8. UV-vis spectra of mCr_2_O_3_ and Au@mCr_2_O_3_-n (n = 0.5, 1.0, 3.0).

Figure S9. a, b) XPS spectra of Au@mCr_2_O_3_-1.0. a) Cr 2*p* spectrum shows two peaks, attributed to the spin-orbitals of Cr *2p_3/2_* and Cr *2p_1/2_*. b) Au 4*f* spectrum exhibits two peaks, which belong to Au 4f_7/2_ and Au 4f_5/2_, respectively.

Figure S10 EPR spectrum of mCr_2_O_3_ and Au@mCr_2_O_3_-n (n = 0.5, 1.0, 3.0).

Figure S11. Mass spectrum of indigo using mCr_2_O_3_, Au@mCr_2_O_3_-0.5, Au@mCr_2_O_3_-1.0, and Au@mCr_2_O_3_-3.0 as matrices, respectively (M represents indigo, laser energy is 80%).

Figure S12. Relationship between laser energy and peak intensity of indigo using Au@Cr_2_O_3_-1.0 as matrix material. Quantitative data are presented as mean ± standard deviation (SD) derived from five independent replicates.

Figure S13. Infrared thermal images of mCr_2_O_3_, Au@mCr_2_O_3_-0.5, Au@mCr_2_O_3_-1.0, and Au@mCr_2_O_3_-3.0.

Figure S14. Mass spectra of BP ions ([BP]^+^ at m/z 170; [BP-pyridine]^+^ at m/z 90) using mCr_2_O_3_, Au@mCr_2_O_3_-0.5, Au@mCr_2_O_3_-1.0, and Au@mCr_2_O_3_-3.0 as matrices, respectively.

Figure S15. a–c) The detection capability of Au@mCr_2_O_3_-1.0 particles as matrix materials for the mixture standard small metabolites solution (a, including Tau, Val, Glc, Asp, Glu, Arg, Phen, and His), in high-salt (b, 0.5 M NaCl) and in high-protein (c, 5 mg/mL BSA) environment. Quantitative data are presented as mean ± standard deviation (SD) derived from three independent replicates.

Figure S16. a, b) Calibration curves for the detection of Glu (a), and Val (b) using Au@mCr_2_O_3_-1.0 as the matrix. The error bars were calculated as the standard deviation (S.D.) of three experiments (n = 3, *p* < 0.05, two-sided *t*-tests).

Figure S17. a–c) The optical images of the matrix-analyte co-crystallization with CHCA (a), DHB (b), and Au@mCr_2_O_3_-1.0 (c).

Figure S18. a–d) The intensities and CVs of DHB (a) and CHCA (b) for detecting Tau, Val, Glc, Asp, and Glu in the same spot. The intensities and CVs of DHB (c) and CHCA (d) for detecting Tau, Val, Glc, Asp, and Glu in the different spots.

Figure S19. Background peaks for different matrices at a m/z of 100~1000. DHB and CHCA exhibit significant noise background, which would cover small metabolite peaks.

Figure S20 (a) SEM image of D-Au@mCr_2_O_3_-1.0. (b) The detection capability of D-Au@mCr_2_O_3_-1.0, P25, and Au@mCr_2_O_3_-1.0 as matrices materials for detecting TBA ions. Mass spectrum of BP ions ([BP]+ at *m/z* 170; [BP-pyridine]^+^ at *m/z* 90) using D-Au@mCr_2_O_3_-1.0 (c) and P25 as matrices (d). (e) The total intensity of BP ions (black line) and survival yield (SY) of parent ions (red line) desorbed from D-Au@mCr_2_O_3_-1.0, Au@mCr_2_O_3_-1.0 and P25. (f) The detection capability of D-Au@mCr_2_O_3_-1.0, P25, and Au@mCr_2_O_3_-1.0 as matrices materials for Glu, Glc, and His.

Figure S21. Optimization of the matrix concentration. Average MS signal intensities of the adduct peaks for 20 mM (a) His and (b) Glc using varying concentrations of the Au@Cr_2_O_3_-1.0 matrix. Data are presented as mean ± standard deviation (SD) derived from 5 independent technical replicates.

Figure S22. The typical urine and plasma MS spectra in the m/z range of 100–500 Da using Au@mCr_2_O_3_-1.0 (1mg mL^-1^) as matrix.

Figure S23. a–d) The scatter plot of probability in the train cohort by SVM (a), NN (b), Xgboost (c), and RF (d). HC: red dots. PD: blue dots.

Figure S24. a–d) The scatter plot of probability in the test cohort by SVM (a), NN (b), Xgboost (c), and RF (d). HC: red dots. PD: blue dots.

Figure S25. a) Volcano plot of all metabolite feature expression. Red spots represent the up-regulated, and blue spots represent the down-regulated in the PD group. b) Venn diagram of 24 *m/z* features filtered with FC > 2 or FC＜0.5, *p*＜0.05, and ReliefF (Top 80 features).

Figure S26. a) Fold change of biomarkers, including two down-regulated metabolites (blue) and four up-regulated metabolites (purple). b) Heatmap of biomarker signal intensity. The color represents the strength of the *m/z* signal.

Figure S27. a–d) The scatter plot of probability in the train cohort by Xgboost (a), NN (b), SVM (c), and RF (d) based on six biomarkers. HC: red dots. PD: blue dots.

Figure S28. a–d) The scatter plot of probability in the test cohort by Xgboost (a), NN (b), SVM (c), and RF (d) based on six biomarkers. HC: red dots. PD: blue dots.

Figure S29. a–d) The scatter plot of probability in the blind test cohort by Xgboost (a), NN (b), SVM (c), and RF (d) modals. HC: red dots. PD: blue dots.

Figure S30. a–d) The confusion matrix from the blind test set by Xgboost (a), NN (b), SVM (c), and RF (d) models.

Figure S31. Potential metabolic pathways differentially altered of six biomarkers. The color and size of each circle are correlated with the *p*-value and pathway impact.

**Table S1.** The limit of detection (LoD) of Au@Cr_2_O_3_-1.0 (1 mg/mL) as matrix materials for standard metabolites.

| Matrix | LoD (pmol)^a^ | | | | |
| --- | --- | --- | --- | --- | --- |
|  | His | Clu | Glc | Val | Asp |
| Au@Cr_2_O_3_-1.0 | 50 | 50 | 25 | 10 | 25 |

a: The LOD was calculated with an S/N ratio of 3.

**Table S2.** The eight standard metabolites in the mixture solution.

| Small molecule | Chemical Formula | [M+H]^+^ | [M+Na]^+^ | [M+K]^+^ |
| --- | --- | --- | --- | --- |
| Taurine | C_2_H_7_NO_3_S | / | 148.004 | 163.977 |
| Valine | C_5_H_11_NO_2_ | / | 140.068 | / |
| Glutamic | C_5_H_9_NO_4_ | / | 170.042 | / |
| L-arginine | C_6_H_14_N_4_O_2_ | 175.119 | 197.100 | / |
| Histidine | C_6_H_9_N_3_O_2_ | / | 178.058 | / |
| Glucose | C_6_H_12_O_6_ | / | 203.053 | 219.026 |
| Aspartic acid | C_4_H_7_NO_4_ | / | 156.026 | / |
| L-phenylalanine | C_9_H_11_NO_2_ | / | 188.069 | / |

**Table S3.** Clinical characteristics of the PD and HC.

| Category | Female | Male | *p*-value ^a)^ | Age (S.D., year) ^b)^ |
| --- | --- | --- | --- | --- |
| PD (n=132) | 63 | 69 | 0.073 | 42.02 (11.61) |
| HC (n=59) | 36 | 23 |  | 32.08 (4.06) |

a) The *p*-value was calculated by the *χ^2^* test.
b) S.D., standard deviation.

**Table S4.** The diagnostic performance of four machine learning models for PD screening (PD/HC) based on SMFs.

|  |  | AUC  (95%CI) | Accuracy  (95%CI) | Precision  (95%CI) | Recall  (95%CI) | F1 score  (95%CI) |
| --- | --- | --- | --- | --- | --- | --- |
| SVM | train | 0.906  (0.824–0.907) | 0.828  0.764–0.892) | 0.829  (0.765–0.893) | 0.828  (0.764–0.892) | 0.830  (0.766–0.894) |
|  | test | 0.934  (0.851–1.000) | 0.860  (0.770–0.950) | 0.860  (0.770–0.950) | 0.860  (0.770–0.950) | 0.860  (0.770–0.950) |
| NN | train | 0.915  (0.854–0.976) | 0.828  (0.764–0.892) | 0.831  (0.768–0.894) | 0.828  (0.764–0.892) | 0.836  (0.773–0.899) |
|  | test | 0.920  (0.829–1.000) | 0.807  (0.705–0.909) | 0.808  (0.706–0.910) | 0.807  (0.705–0.909) | 0.810  (0.708–0.912) |
| Xgboost | train | 0.926  (0.868–0.984) | 0.821  (0.756–0.886) | 0.817 (0.752–0.882) | 0.821  (0.756–0.886) | 0.816  (0.750–0.882) |
|  | test | 0.923  (0.834–1.000) | 0.842  (0.747–0.937) | 0.841  (0.746–0.936) | 0.842  (0.747–0.937) | 0.840  (0.745–0.935) |
| RF | train | 0.902  (0.836–0.968） | 0.806  (0.739–0.873) | 0.803  (0.736–0.870) | 0.806  (0.739–0.873) | 0.802  (0.735–0.869) |
|  | test | 0.875  (0.764–0.970) | 0.754  (0.642–0.866) | 0.745  (0.632–0.858) | 0.754  (0.642–0.866) | 0.745  (0.632–0.858) |

**Table S5.** Information of metabolic biomarkers.

| ID | *m/z* | Metabolite | Adduct type | Chemical Formula | HMDB ID | FDR |
| --- | --- | --- | --- | --- | --- | --- |
| ID24 | 111.2079 | Putrescine | M+Na | C_4_H_12_N_2_ | HMDB0001414 | ＜0.01 |
| ID51 | 128.7789 | Lactic acid | M+K | C_3_H_6_O_3_ | HMDB0000190 | ＜0.01 |
| ID57 | 131.8522 | Creatine | M+H | C_4_H_9_N_3_O_2_ | HMDB0000064 | ＜0.01 |
| ID68 | 139.2573 | Fumaric acid | M+Na | C_4_H_4_O_4_ | HMDB0000134 | ＜0.01 |
| ID95 | 157.8536 | Homocysteine | M+Na | C_4_H_9_NO_2_S | HMDB0000742 | ＜0.01 |
| ID294 | 353.8091 | 17-Hydroxyprogesterone | M+Na | C_21_H_30_O_3_ | HMDB0000374 | ＜0.01 |

**Table S6.** The diagnostic performance of four machine learning models based on metabolic biomarkers in identifying PD.

|  |  | AUC  (95%CI) | Accuracy  (95%CI) | Precision  (95%CI) | Recall  (95%CI) | F1 score  (95%CI) |
| --- | --- | --- | --- | --- | --- | --- |
| SVM | train | 0.851  (0.799–0.914) | 0.799  (0.731–0.867) | 0.791  (0.722–0.860) | 0.799  (0.731–0.867) | 0.791  (0.722–0.860) |
|  | test | 0.708  (0.555–0.861) | 0.737  (0.623–0.851) | 0.735  (0.620–0.850) | 0.737  (0.623–0.851) | 0.733  (0.618–0.848) |
| NN | train | 0.879  (0.807–0.951) | 0.791  (0.722–0.860) | 0.790  (0.721–0.859) | 0.791  (0.722–0.860) | 0.788  (0.719–0.857) |
|  | test | 0.829  (0.702–0.956) | 0.737  (0.623–0.851) | 0.744  (0.631–0.857) | 0.737  (0.623–0.851) | 0.762  (0.652–0.872) |
| Xgboost | train | 0.900  (0.834–0.966) | 0.821  (0.756–0.886) | 0.821  (0.756–0.886) | 0.821  (0.756–0.886) | 0.821  (0.756–0.886) |
|  | test | 0.933  (0.849–1.000) | 0.895  (0.815–0.975) | 0.898  (0.820–0.976) | 0.895  (0.815–0.975) | 0.921  (0.851–0.991) |
| RF | train | 0.915  (0.869–0.961) | 0.858  (0.799–0.917) | 0.859  (0.800–0.918) | 0.858  (0.799–0.917) | 0.859  (0.800–0.918) |
|  | test | 0.957  (0.909–1.000) | 0.860  (0.770–0.950) | 0.887  (0.805–0.969) | 0.860  (0.770–0.950) | 0.864  (0.775–0.953) |

**Table S7.** Summary of metabolic pathways regulated in PD patients and HC.

| Pathway | -log(p) | FDR | Impact |
| --- | --- | --- | --- |
| Arginine and proline metabolism | 2.1520 | 0.2819 | 0.2116 |
| Cysteine and methionine metabolism | 0.9271 | 0.9462 | 0.1382 |
| One carbon pool by folate | 1.0259 | 0.9462 | 0.1039 |
| Steroid hormone biosynthesis | 0.5427 | 1 | 0.0962 |
| Citrate cycle (TCA cycle) | 1.1357 | 0.9462 | 0.0298 |
| Tyrosine metabolism | 0.8282 | 1 | 0.02463 |
| Glutathione metabolism | 0.9951 | 0.9462 | 0.0072 |
| Alanine, aspartate and glutamate metabolism | 0.9951 | 0.9462 | 0.0024 |
| Glycine, serine and threonine metabolism | 0.9271 | 0.9462 | 0 |
| Glycolysis or Gluconeogenesis | 1.0259 | 0.9462 | 0 |
| Pyruvate metabolism | 2.5387 | 0.2314 | 0 |
| Arginine biosynthesis | 1.2865 | 0.9662 | 0 |
